# Cancer incidence in Rabat, Morocco: 2006–2008

**DOI:** 10.3332/ecancer.2013.338

**Published:** 2013-08-08

**Authors:** Mohammed Adnane Tazi, Abdelouahed Er-Raki, Noureddine Benjaafar

**Affiliations:** Institut National d’Oncologie, Av Allal El Fassi, BP 6213, Rabat 10100, Morocco

**Keywords:** cancer, incidence, population-based registry, Rabat, Morocco

## Abstract

**Introduction:**

No population-based data of cancer incidence from Morocco have been published before. This is the first report of cancer incidence in Rabat from a population-based cancer registry for the period 2006–2008.

**Materials and methods:**

The cancer registry collects data on all new cases of cancer diagnosed in the resident population of the Rabat area. Data collection is an active process involving visits by registry staff to all data sources, essentially hospitals, pathological laboratories, and private clinics in Rabat.

**Results:**

A total of 2,473 new cases of cancer were registered among residents in Rabat during the period 2006–2008. The overall world age-standardised rate (ASR) for all sites combined was 136.6/100,000 for men and 114.5/100,000 for women. The most frequently diagnosed malignancies in males were lung cancer (19.0%, ASR 24.8/10^5^), followed by prostate cancer (15.5%, ASR 22.9/10^5^), colorectal cancer (8.8%, ASR 12.0/10^5^), bladder cancer (6.9%, ASR 9.7/10^5^), and non-Hodgkin’s lymphoma (NHL) (6.0%, ASR 8.2/10^5^). In females, the most frequently reported malignancies were breast cancer (39.9%, ASR 43.4/10^5^), followed by cervix uteri cancer (11.4%, ASR 13.0/10^5^), colorectal cancer (7.5%, ASR 9.0/10^5^), NHL (3.4, ASR 4.2/10^5^), and thyroid cancer (3.4%, ASR 3.9/10^5^).

Of all cancers, 2% are observed in childhood (0–14 years), and ~43% of them are malignant haemopathies.

**Conclusion:**

The data reported by Rabat Cancer Registry indicate that cancers of the breast, cervix, uteri, and colon and rectum in females and the lung, prostate, and colon and rectum in men are major cancers in the population of Rabat. The Incidences observed are sometimes different from those observed in the neighbouring North African countries.

## Introduction

Morocco, a North African country, has not had a population-based cancer registry for long, although many attempts have been made since 1990 to establish a cancer registry in the Rabat region, due to the lack of human and material resources.

In January 2005, as a part of a cooperative project between the Moroccan Ministry of Health and the World Health Organization, a population-based cancer registry was created in Rabat, and the first report concerning the data of 2005 has already been published [1]. Rabat— the Moroccan capital—has a well-developed health-care delivery system. Cancer care is provided by five university hospitals, the National Institute of Oncology, the military hospital, two private cancer centres, and many private clinics. There are five linear accelerators in public hospitals and four in private centres.

Morocco has been adopting, for the period 2010–2019, the National Plan for Prevention and Cancer Control (NPPCC). Cancer is a major public health problem and is responsible for 56% of morbidity related to chronic diseases in Morocco. The NPPCC aims to reduce the morbidity and mortality from cancer and to improve the quality of life for patients. The Rabat cancer registry will play a key role in the assessment and guidance of certain actions of the NPPCC.

It is well known that the data provided by cancer registries during their early years of operation are certainly interesting but the data are subject to improvement during the following years, becoming more reliable and allowing better determination of the real burden of cancer and the differences from other registries.

In this paper, we report the data over three years (2006–2008) of the Rabat registry’s activity.

## Material and Methods

The Rabat population-based cancer registry covers all the incidences of cancer cases from 2005 onward among residents in Rabat.

**Figure 1:**
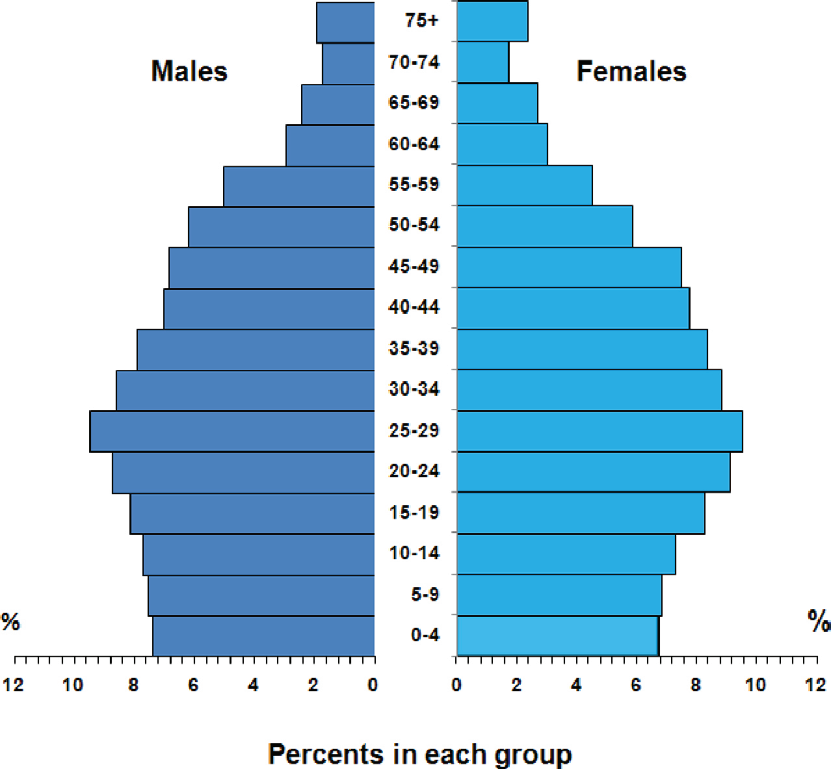
The average annual population at risk, Rabat 2006–2008.

Rabat, Morocco’s political capital, is located on the country’s northwest Atlantic coast. It is an administrative city; all inhabitants are urban, and almost all are Muslims. The total population is 642,000 (51.8% males and 48.2% females) according to 2007 estimates. The median age in Rabat is higher than the overall Moroccan population, and the pyramid of ages is different, with 21.7% children (0–14 years) and 9.5% people older than 60 years (in the Moroccan population: 29.1% are under 15 years, and 8.0% are over 60) (Figure 1).

The collection of registry data is an active process involving visits by registry staff to 65 different locations (laboratories, hospitals, private clinics, etc). The data sources are all the public or private facilities in Rabat in which cancer may be diagnosed or treated, essentially the pathology laboratories, the National Institute of Oncology, University Hospital Ibn Sina, private cancer centres, private clinics, Military Hospital Mohammed V, and the offices of some private practitioners (in particular, haematologists). For blood cancers, case data are also collected at the haematology–oncology service of Ibn rochd University Hospital in Casablanca, which is a specialised centre for the treatment of blood cancers. Death certificates were not considered because they were of inadequate quality because they often provide an inaccurate cause of death and it is difficult to access the registry of deaths.

The data reported in this analysis concern the new cancer cases of malignant tumours diagnosed during the period from 1 January 2006 to 31 December 2008, for residents in the geographical area covered by the registry.

The cases registered included invasive cancers, *in situ *lesions, and borderline tumours in all anatomical sites (including nonmelanoma skin cancers), but only invasive cancers are considered in the calculation of the incidence rates. The registrations are considered microscopically verified when the diagnosis is based on a malignant histological or cytological report.

The cases were classified according to the third edition of the International Classification of Disease for Oncology (ICD-O-3), and coding practices (including the basis and date of diagnosis) were defined according to the current international guidelines [2].

The data collection and coding were done by resident physicians in radiotherapy at the National Institute of Oncology. Before being sent to abstract cancer cases, the residents had *ad hoc *training about the mission of the Cancer Registry, the general rules for the registration of cases, and technical data coding cancer. The investigators extract data from patient medical records and from the pathology records. The data entry is done by the registry staff trained for this task.

To avoid recording duplicate cases from different information sources, a careful comparison of the data is made between certain variables, such as name, age, national identity number, telephone number, cancer topography and morphology, and so forth. To check the internal consistency (the validity) of the data, we searched for aberrations or incompatibilities between different variables in the same record, such as age/topography, topography/morphology, sex/topography, and so forth. This monitoring was carried out mainly using the tool of the International Association of Cancer Registries, the IARCcrgTools program.

The proportion of cases without information for some variables (age, sex, and primary site) is another criterion used for assessing the quality of the data. The age is unknown for 0.7% only, the sex is known for all cases, and the primary site is known for 96.6%.

The results are presented as the number of cases by site (ICD-10), sex, and age, with crude, age-specific, and age-standardised incidence rates (ASRs) per 100,000. The calculation of the ASR is carried out by direct methods, using the world standard population, and the cumulative risk of developing cancer before the age of 75 (CR_0–74_) is estimated according to the methodology proposed by the IARC [3]. The person-years of the population at risk by sex and five-year age groups (Figure 1) were obtained from ‘the High Commissariat for Planning,’ an official structure in Morocco, which gives an annual estimation of the population size for the years intercensus (last census was in 2004) by gender, age groups, and region [4].

The software Epi-Info is used for data entry, and Microsoft Excel and SPSS are used for statistical analysis.

## Results

A total of 2,473 new cancer cases (1,241 in males and 1,232 in females) were registered in Rabat during the period between January 2006 and December 2008. The median age at time of diagnosis was 62 years for males and 54 years for females.

**Figure 2:**
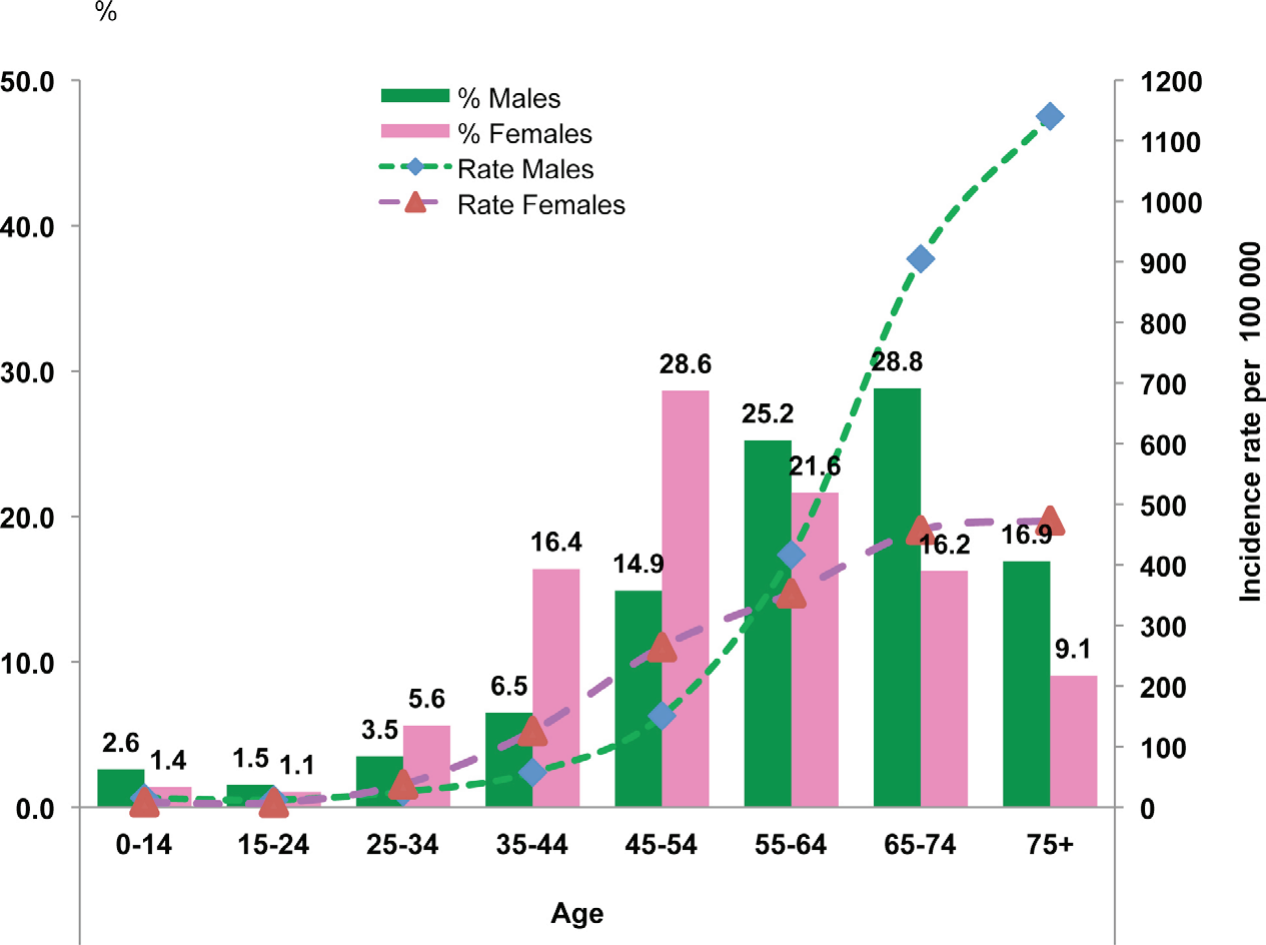
The age distribution (%) and age-specific incidence rates for all cancers combined, Rabat, 2006–2008.

The distribution of cases and the crude incidence rate by age group and sex are shown in Figure 2. The cancer incidence among both sexes increases with age and is higher among women between 35 and 55 years and higher in men after age 65.

The percentage of pathological confirmation is very high for most sites (98% overall), except for the pancreas and liver, sites that are difficult to access (Table 1).

The total number of cases by site, the percentage distribution, age-specific rates, crude rate and ASR for males and females are given in Tables 2 and 3. The ASR for all cancers combined was 136.6/100,000 males and 114.5/100,000 females, and the CR_0–74 _is 15.2% and 12.1% for males and females, respectively. The ASR for all cancers combined was 136.6/100,000 males and 114.5/100,000 females, and the CR_0–74 _is 15.2% and 12.1% for males and females, respectively. The most frequently reported cancer sites in males were the lungs (19.0% of all cases, ASR: 24.8, CR_0–74_: 3.0%), prostate (15.5%, ASR: 22.9, CR_0–74_: 3.1%), colon and rectum (8.8%, ASR: 12.0, CR_0–74_: 1.5%), bladder (6.9%, ASR: 9.7, CR_0–74_: 1.2%), NHL **= **(6.0%, ASR: 8.2, CR_0–74_: 0.9%), and stomach (3.9%, ASR: 5.0, CR_0–74_: 0.6%). In females, the most common site of cancers was the breasts (39.9%, ASR: 43.4, CR_0–74_: 4.5%), followed by cervix uteri (11.4%, ASR: 13.0, CR_0–74_: 1.6%), colon and rectum (7.5%, ASR: 9.0, CR_0–74_: 1.1%), NHL (3.4, ASR: 4.2, CR_0–74_: 0.5%), thyroid (3.4%, ASR: 3.9, CR_0–74_: 0.4%), and ovaries (2.8%, ASR: 3.2, CR_0–74_: 0.4%).

**Figure 3:**
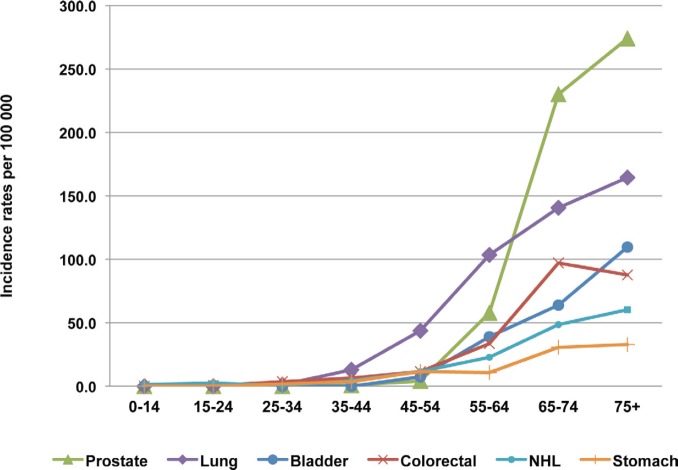
The age-specific incidence rate for prostate, lung, bladder, colorectal, and stomach cancers and NHL in males, Rabat, 2006–2008.

**Figure 4:**
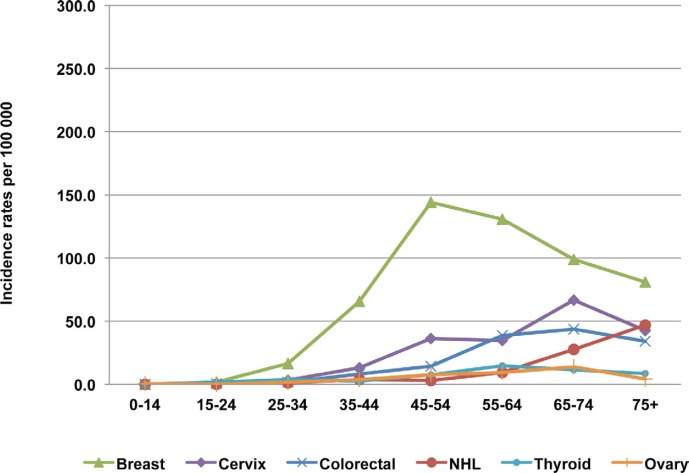
The age-specific incidence rate for breast, cervix uteri, colorectal, ovarian, and thyroid cancers and NHL in females. Rabat, 2006–2008

The age-specific incidence rates of the six most frequent sites among males are shown in Figure 3. The Incidence rate increases sharply with age after 65 years. After 75 years, the incidence reaches 274/100,000 for prostate cancer and 164/100,000 for lung cancer. In females, the incidence rate of the most frequent cancers increases with age, especially for cervical cancer after 45 years and for colorectal cancer after 50 years, but for breast cancer, the incidence peak is between 45 and 55 years, and the incidence decreases gradually after this age (Figure 4).

Skin cancer is uncommon before the age of 65; its incidence is quite high in males and increases with age after the age of 65. It is often a basal cell carcinoma (38.9%) or squamous cell carcinoma (31.9%).

In Rabat, 271 malignant haemopathies were recorded between 2006 and 2008, 11% of all cases, and their incidence rate is significantly higher in men than in women (ASR: 18.2 versus 10.5/100,000). The most frequent is NHL (43%), which comes in the fifth position in males and in fourth in females, followed by leukaemia (23%). The NHL is nodal in 43% of cases, while the most frequent extranodal sites are the stomach (12%) and nasopharynx (6%). Nearly half of the NHL cases are large B-cell lymphoma.

Overall, cancers in children aged 0–14 years in Rabat represent 2% of all cancers, and their incidence is twice as high among males than females (15.2 versus 8.2/100,000 children). About 43% of cancers in childhood are malignant haemopathies (leukaemia: 29%, lymphoma: 10% and others: 4%), and the other frequent localisations are the brain (18%), kidney (14%), and eye (8%).

The ASRs of the major cancer sites in males and females from some population-based cancer registries in North African countries are given in Table 4. In Rabat, the ASR seems to be higher for prostate cancer and cervix cancer, but in general for the major sites, the incidence of cancer in Rabat seems to be similar to those observed in other North African registries.

## Discussion

The cancer incidence data from a well-defined population of Rabat are reported. The Rabat population size, by age group and gender, covered by the cancer registry is reliable because it is given by an official structure of the Moroccan state, the ‘High Commission for Planning,’ which provides an annual estimation of the population for each region in Morocco.

The data collected were generally valid, according to the standard registration practices followed by cancer registries around the world [3]. The data collection and coding were done by resident physicians in oncology; it is certain that this has reduced some potential misclassification enormously, especially in coding the morphology and topography. The data were collected from multiple data sources, and the information was regularly and carefully scrutinized. Extreme attention has been paid to eliminating duplicate cases collected from more than one source, and prevalent cases diagnosed in the years before the registration period are reported here.

The percentage of cancers histologically confirmed in the Rabat registry (98%) is similar to the percentages observed in Western countries’ registries and even to those in neighbouring countries, but it remains high compared with those observed in many other cancer registries in Africa and in developing countries in general [5–9]. This can be explained initially by the widely available diagnosis facilities in Rabat and the general attitude of the physicians to use all available means to gather histological evidence of cancer and secondarily by nonregistration of cases by the registry team without histological evidence or sufficient arguments about the malignancy. However, in outpatient departments or in clinicians’ offices, cases in advanced malignancy stages might be only clinically diagnosed and less likely to be subjected to intense diagnostic and therapeutic procedures. Consequently, some of these might not be registered in hospitals and clinics, so there is the possibility of under registration, especially since we could not yet use the death certificate notification as an information source. It is difficult to assess the bias related to the lack of cases recorded from death certificate only (DCO), but we estimate that the bias introduced on the incidence measurements would be minimal, given the high-quality medical care on offer and the easy access to care in Rabat. According to the data of some similar cancer registries, cases reported by DCO vary between 0.2% and 3.3% [10–13].

We have obtained data from pathology laboratories for the majority of cases. So, date of diagnosis was in general precisely defined, and we have more information about primary site and histological type that reduce the risk of misclassification or recording prevalent cases.

We observed a low proportion of unknown primary sites in our data like other records in other registries in developed countries [14, 15]. This is probably due to the availability of good medical infrastructure in Rabat with widely available diagnostic facilities, but also to the good cooperation between the registry team and all public and private medical structures and pathology laboratories. This small proportion of unknown primary sites may indicate a good quality of data, but it may be related to an under-registration due, for example, to a nonuse of death certificates as an information source.

For a small number, there was a doubt about the patient address and the case was not recorded. This situation remained rare, and its impact on the cancer incidence must be negligible.

The overall cancer incidence rate and the cancer distribution by site in Rabat and in the other North African countries are quite similar, with a few exceptions [16–20]. The observed similarities are not surprising and are compatible with what is known of the potential risk and protective factors in the region: low industrialisation, control of infectious diseases, Mediterranean diet, religious prohibitions (alcohol, and to a lesser extent tobacco), and sexual and reproductive behaviours [21]. In addition, the overall incidence rate is higher in men than in women, which is the case in most of the cancer registries in the world.

In addition, we note that the incidence rates for both sexes in North Africa are lower than those observed in more developed countries, although the most frequent cancers are more or less the same (lung, breast, colorectal and prostate), unlike the countries of sub-Saharan Africa, where a predominance of cancers is related to infections [8].

In Rabat, two out of five cancers in women are breast cancers, and the most frequent morphology is the ductal subtype (85%). In the last two decades in Morocco, there has been a change in some reproductive factors in women that are known to be associated with a risk of breast cancer: steady increase of the mean age at the first marriage of women (from 17.5 years in 1960 to 26.6 years in 2010) [22], increase of age at first pregnancy, decrease of the synthetic fertility index (from 7.2 in 1962 to 2.3 in 2009), and less breastfeeding [23, 24]. Thus, it is possible that those factors associated with a change in lifestyle and dietary habits have been associated with the increased risk of breast cancer. Breast cancer is now the most frequent cancer site among females in Rabat. This incidence rate is closer to the average rates reported by cancer registries in North Africa and in other developing countries but remains lower than the rates observed in Europe and North America [9, 12, 14, 25–28]. Incidence is highest in the age group 45–64 and it seems that in Morocco, as in many other African countries, breast cancer affects more women at a younger age than in the European and American population. So, in Morocco, such relatively low incidence and low average age make the screening programmes of breast cancer commonly used in Western countries less effective.

The incidence of lung cancer, in Morocco as in the other North African countries, is high among males, certainly because of the high prevalence of smoking. According to a national survey conducted in Morocco in 2006 among people aged between 15 and 75 years old, the smoking prevalence is 31% in males [29]. In females, where the smoking prevalence is very low (3%), lung cancer is ten times less frequent. However, the incidence rate of lung cancer remains low compared with those rates reported in other countries in Europe, North America and the Far East. According to the recent surveys realised by the Moroccan Ministry of Health, there is an increase in schoolchildren starting a smoking habit [30, 31]. Lung cancer and other smoking-related cancers may increase in the future if proper intervention is not implemented to fight smoking. Moreover, the low incidence of lung cancer in women indicates that it is unlikely that lung metastases of other sites have been incorrectly classified as a lung cancer.

Although prostate cancer incidence in Rabat is less than in developed countries’ populations, it remains relatively high, compared with the rate in neighbouring Maghreb countries [16–18, 20]. The incidence of prostate cancer is often influenced by the prostate-specific antigen control in the elderly by physicians (mass programme screening or individual screening) [32]. In Rabat, the socioeconomic level is relatively high compared with the rest of Morocco, and a significant proportion of the population has health insurance. So, it seems that there is a wide availability of individual screening in Rabat and that such practice contributes to this relatively high incidence of prostate cancer.

The incidence rate of bladder cancer, compared with that observed in some European countries and in the United States, is relatively low in Morocco and in the other North African countries, except in Egypt where the estimated incidence rate in males is the highest in the world [33]. In general, bladder cancer, most commonly a transitional cell carcinoma (TCC), is more related to cigarette smoking, whereas in Egypt, the highest incidence rate of bladder cancer, most frequently a squamous cell carcinoma (SCC), was related to the high prevalence of schistosomiase, but recently, TCC incidence has increased, while SCC has declined.

Colorectal cancer is the most common gastrointestinal tract cancer for both sexes in Rabat, but its incidence rate is closer to the rates in the North African and Middle East countries, and it remains lower than in Western countries or the Far East. This relatively low incidence is probably related to a Mediterranean lifestyle in these countries.

Stomach cancer is particularly common in the populations of some countries in the Far East, such as Japan, and South America, while its incidence is relatively low in Maghreb and Africa in general.

Thyroid cancer is relatively less common in North African countries compared with other regions of the world, including Western Europe and North America, whereas nasopharyngeal cancer is common in the Maghreb population, where Algeria and Morocco are considered an intermediate incidence area between the Chinese population and Western populations.

The incidence rates of malignant haemopathies, particularly NHL and leukaemia, are close to the incidences observed in the other Maghreb registries, but they are less than those observed in Western countries.

Figures shown in this paper give an accurate description of the cancer incidence in the Rabat area and reinforce the observation that in Morocco, the epidemiology of cancer has many similarities and some differences with the other North African countries, but the risk of developing cancer is largely lower in the North African region than in most other regions in the world. This is a favourable element for health policy in our country, which, less crowded than others by the amount of cases, may invest more and better in the development of the quality of provision of prevention and care.

The patterns observed from the analysis of the Rabat data provide a valuable tool for cancer control in Morocco. They indicate what the most common cancers are and what the priorities are. Awareness campaigns and screening programmes are needed to fight against the most common cancers to reduce the burden of morbidity and mortality from cancer in Morocco. It is noteworthy that tobacco control measures are necessary to reduce the incidence of tobacco-related cancers, and for breast cancer, which is the most frequent cancer in females, an early detection linked with treatment should be beneficial in controlling this disease. In addition, research on cancer should be developed to improve our understanding of this disease and its major risk factors. The incidence rate might increase in the coming years due to the ageing of the population and also because of the increasing westernisation of the lifestyle in Morocco. Otherwise, it is important to develop other population-based cancer registries in other Moroccan regions to improve even more our knowledge of cancer epidemiology in Morocco and to adapt, if necessary, our national strategy to fight cancer.

## Acknowledgments

The authors would like to thank the resident physicians in radiotherapy at the National Institute of Oncology for their contribution in data collection and coding, as well as all the staff responsible for the private and public medical structures to have facilities to access their data and pathology laboratories in Rabat, the haematology–oncology service in the University Hospital of Casablanca, and the medical staff of the military hospital, the Director of the High Commission for Planning, the Director of the Studies and Demographic Research Centre for their help and cooperation with the registry team. The support provided by the ‘Rabat Cancer Registry Committee’ and Prof. Roberto Zanetti, Director of Piedmont cancer registry is gratefully acknowledged. The authors also thank Mr. Tarik Boudaka for his help in proofreading in English

## Figures and Tables

**Table 1.** The percentage of cases with pathological verification by sex in Rabat, 2006–2008.

Site	Males (%)	Females (%)
All sites	97	99
Nasopharynx	100	100
Oesophagus	100	100
Stomach	100	100
Colon	97	100
Rectum	100	100
Liver	83	87
Gallbladder and biliary tract	95	100
Pancreas	63	72
Larynx	100	100
Lung	95	100
Breast (females)	–	100
Cervix uteri	–	100
Corpus uteri	–	100
Ovary	–	97
Prostate	99	–
Bladder	100	100
Kidney	100	100
Brain and nervous system	94	94
Thyroid	100	100

**Table 2.** The relative frequency (%), age-specific incidence rates, average annual crude incidence rates, and ASR by site in males in Rabat, 2006–2008.

Localisation	All ages	%	0–14	15–24	25–34	35–44	45–54	55–64	65–74	75+	Crude rate	ASR world	ICD-10
Oral cavity	27	2.2	0.0	0.0	0.6	0.7	4.1	16.1	12.8	11.0	2.9	2.9	C00–08
Nasopharynx	23	1.9	0.5	0.0	4.2	4.3	0.0	5.4	7.7	11.0	2.5	2.3	11
Other pharynx	9	0.7	0.0	0.6	0.6	0.0	2.5	1.3	5.1	5.5	1.0	0.9	C09–10 and 12–13
Oesophagus	19	1.5	0.0	0.0	0.0	1.4	0.0	10.8	15.3	16.4	2.0	2.1	15
Stomach	48	3.9	0.0	0.0	1.8	3.6	11.6	10.8	30.7	32.9	5.2	5.0	16
Colon and rectum	109	8.8	0.0	0.6	3.6	6.5	11.6	33.6	97.2	87.7	11.8	12.0	C18–20
Liver	18	1.5	0.0	0.0	0.6	0.0	0.0	4.0	20.5	32.9	1.9	2.1	C22
Pancreas	30	2.4	0.0	0.0	0.0	0.7	1.7	12.1	28.1	38.4	3.2	3.6	25
Larynx	34	2.7	0.0	0.0	0.0	2.2	2.5	13.4	25.6	38.4	3.7	3.7	32
Bronchus and lungs	237	19.1	0.0	0.0	1.2	13.0	43.7	103.5	140.6	169.9	25.6	24.9	C33–34
Bone	9	0.7	0.5	1.9	0.6	0.0	0.8	2.7	2.6	0.0	1.0	0.9	C40–41
Malignant skin melanoma	0	0.0	0.0	0.0	0.0	0.0	0.0	0.0	0.0	0.0	0.0	0.0	43
Other malignant skin	41	3.3	0.0	0.0	0.0	0.7	6.6	4.0	33.2	76.8	4.4	4.3	44
Connective and soft tissue	9	0.7	0.0	0.0	1.2	0.0	0.8	2.7	7.7	5.5	1.0	0.9	49
Breast	9	0.7	0.0	0.0	0.0	0.0	2.5	4.0	2.6	11.0	1.0	0.9	50
Prostate	192	15.5	0.0	0.0	0.0	0.7	4.1	57.8	230.1	274.1	20.7	22.9	61
Testis	10	0.8	0.5	0.6	1.8	2.2	0.8	1.3	0.0	0.0	1.1	1.0	62
Bladder	86	6.9	0.0	0.6	0.0	0.0	7.4	39.0	63.9	109.6	9.3	9.7	67
Kidney	21	1.7	2.8	0.0	0.0	0.7	1.7	5.4	7.7	27.4	2.3	2.7	C64–66 and C68
Brain and nervous system	34	2.7	2.4	0.0	1.8	4.3	4.1	9.4	17.9	5.5	3.7	3.7	C70–72
Gland thyroid	10	0.8	0.0	0.0	0.6	2.2	2.5	2.7	0.0	5.5	1.1	0.9	73
Hodgkin’s disease	23	1.9	0.0	3.8	0.6	2.2	5.8	1.3	7.7	11.0	2.5	2.3	C81
Non-Hodgkin’s lymphoma	75	6.0	1.4	2.6	0.6	4.3	11.6	22.9	48.6	60.3	8.1	8.2	C82–85 and C96
Myeloma	25	2.0	0.0	0.0	0.0	0.0	5.0	6.7	15.3	38.4	2.7	2.6	C90
Lymphoid leukaemia	16	1.3	2.8	0.0	1.2	0.0	0.8	4.0	5.1	11.0	1.7	2.1	C91
Myeloid leukaemia	18	1.5	0.9	1.3	1.8	2.2	1.7	4.0	5.1	0.0	1.9	1.9	C92
Others	109	8.8	3.3	0.0	3.0	5.8	17.3	37.7	74.1	60.3	11.8	12.2	–
**All sites**	**1241**	**100**	**15.2**	**12.1**	**25.6**	**57.6**	**151.0**	**416.8**	**905.1**	**1140.3**	**133.8**	**136.6**	–
All sites but skin	1200	96.7	15.2	12.1	25.6	56.9	144.4	412.8	871.9	1063.5	129.4	132.3	–

**Table 3.** The relative frequency (%), age-specific incidence rates, average annual crude incidence rates, and ASR by site in females in Rabat, 2006–2008.

Localisation	All ages	%	0–14	15–24	25–34	35–44	45–54	55–64	65–74	75+	Crude rate	ASR world	ICD-10
Oral cavity	15	1.2	0.0	0.0	0.5	1.2	0.8	8.0	4.6	12.8	1.5	1.5	C00–08
Nasopharynx	16	1.3	0.0	0.0	1.1	3.7	4.5	1.3	2.3	0.0	1.6	1.3	11
Other pharynx	3	0.2	0.0	0.0	0.5	1.2	0.0	0.0	0.0	0.0	0.3	0.2	C09–10 and 12–13
Oesophagus	10	0.8	0.0	0.0	0.5	0.0	3.8	1.3	0.0	12.8	1.0	0.9	15
Stomach	33	2.7	0.0	0.6	0.0	3.1	6.0	9.3	20.7	12.8	3.3	3.2	16
Colon and rectum	92	7.5	0.0	0.6	1.6	8.1	14.3	38.7	43.7	34.1	9.2	9.0	C18–20
Liver	15	1.2	0.0	0.0	0.0	0.0	0.8	0.0	20.7	21.3	1.5	1.5	C22
Pancreas	18	1.5	0.0	0.0	0.5	0.0	2.3	5.3	18.4	8.5	1.8	1.9	25
Larynx	5	0.4	0.0	0.0	0.0	0.6	0.0	4.0	2.3	0.0	0.5	0.5	32
Bronchus and lungs	27	2.2	0.0	0.0	0.5	2.5	3.0	13.3	9.2	17.0	2.7	2.6	C33–34
Bone	2	0.2	1.0	0.0	0.0	0.0	0.0	0.0	0.0	0.0	0.2	0.2	C40–41
Malignant skin melanoma	6	0.5	0.0	0.0	0.0	0.6	0.0	1.3	9.2	0.0	0.6	0.6	43
Other malignant skin	25	2.0	0.0	0.0	0.0	2.5	0.8	1.3	6.9	59.7	2.5	2.0	44
Connective and soft tissue	6	0.5	0.0	0.6	0.0	1.9	0.0	1.3	2.3	0.0	0.6	0.6	49
Breast	491	39.9	0.0	1.7	16.4	65.6	144.1	130.7	98.9	81.0	49.2	43.4	50
Cervix	140	11.4	0.0	0.0	3.3	13.1	36.2	34.7	66.7	42.6	14.0	13.0	53
Ovary	34	2.8	0.5	0.0	1.6	3.7	7.5	9.3	13.8	4.3	3.4	3.2	56
Bladder	7	0.6	0.0	0.0	0.0	0.0	0.0	2.7	4.6	12.8	0.7	0.7	67
Kidney	17	1.4	0.5	0.0	0.5	0.6	5.3	4.0	2.3	12.8	1.7	1.6	C64–66 and C68
Brain and nervous system	16	1.3	1.4	0.0	0.5	1.2	1.5	8.0	4.6	0.0	1.6	1.7	C70–72
Gland thyroid	42	3.4	0.0	1.7	3.8	2.5	7.5	14.7	11.5	8.5	4.2	3.9	73
Hodgkin’s disease	15	1.2	1.0	1.7	1.6	0.6	0.8	1.3	2.3	8.5	1.5	1.3	C81
Non-Hodgkin’s lymphoma	42	3.4	0.0	0.0	1.1	3.7	3.0	9.3	27.6	46.9	4.2	4.2	C82–85 and C96
Myeloma	13	1.1	0.0	0.0	0.0	0.0	1.5	5.3	9.2	12.8	1.3	1.3	C90
Lymphoid leukaemia	7	0.6	1.4	0.0	0.0	0.0	1.5	1.3	2.3	0.0	0.7	0.9	C91
Myeloid leukaemia	19	1.5	1.4	0.0	1.1	1.9	3.0	4.0	4.6	8.5	1.9	1.8	C92
Others	116	9.4	1.0	0.6	2.2	6.9	16.6	42.7	69.0	55.4	11.6	11.3	–
**All sites**	**1232**	**100**	**8.2**	**7.5**	**37.8**	**125.6**	**264.7**	**353.4**	**457.5**	**473.0**	**123.5**	**114.5**	–
All sites but skin	1207	98.0	8.2	7.5	37.8	123.1	264.0	352.1	450.6	413.3	121.0	112.5	–

**Table 4.** The world ASR in Rabat compared to other North African countries.

	Rabat, Morocco (2006-2008)	Setif, Algeria (2006-2008)	Tunisia—North (2004-2006)	Benghazi, Libya (2004)	Aswan, Egypt(2008)
**Site**					
**Males**					
Oral Cavity	2.9	NA	2.8	0.95	4.9
Nasopharynx	2.3	5.4	3.6	4.0	1.1
Oesophagus	2.1	NA	0.6	1.2	5.7
Stomach	5.0	8.1	6.1	4.5	4.1
Colon and rectum	12.0	8.8	11.6	14.3	5.0
Pancreas	3.6	NA	2.9	6.2	5.7
Larynx	3.7	9.7	5.6	5.3	6.0
Bronchus and lung	24.9	23.8	32.5	26.7	11.2
Skin (excluding melanoma)	4.3	NA	7.7	2.7	1.8
Breast	0.9	NA	0.6	0.5	1.8
Prostate	22.9	7.4	11.8	9.8	9.2
Bladder	9.7	10.4	13.7	12.6	18.6
Kidney	2.7	NA	2.6	3.8	1.3
Brain, nervous system	3.7	NA	2.8	4.6	6.3
Gland thyroïd	0.9	NA	1.0	1.0	1.1
Hodgkin’s disease	2.3	NA	2.2	2	1.7
Non-Hodgkin’s lymphoma	8.2	7.2	5.5	6.4	2.2
Myeloma	2.6	NA	0.9	1.9	0.3
Lymphoid leukaemia	2.1	NA	1.7	1.5	3.7
Myeloid leukaemia	1.9	NA	1.4	3.2	2.5
**All sites**	**136.6**	**123.3**	**133.2**	**129.5**	**142.5**
					
**Females**					
Oral Cavity	1.5	NA	1.5	1.8	2.2
Nasopharynx	1.3	0.9	1.5	1.4	0.2
Oesophagus	0.9	NA	0.4	0.3	1.6
Stomach	3.2	3.4	3.7	2.1	3.4
Colon and rectum	9.0	11.3	9.5	12.2	4.8
Pancreas	1.9	NA	1.4	3.9	2.3
Larynx	0.5	NA	0.3	0.4	0.7
Bronchus and lung	2.6	NA	2.9	2.0	3.8
Skin (excluding melanoma)	2.0	NA	5.6	1.8	2.8
Breast	43.4	24.8	31.8	23.3	63.9
Cervix	13.0	9.2	4.2	3.5	0.9
Ovary	3.2	NA	4.3	3.9	9.1
Bladder	0.7	NA	1.3	3.8	6.6
Kidney	1.6	NA	1.6	1.3	1.1
Brain, nervous system	1.7	NA	2.3	3.3	2.8
Gland thyroïd	3.9	7.2	3.3	3.9	4.5
Hodgkin’s disease	1.3	NA	1.4	1.3	0.8
Non-Hodgkin’s lymphoma	4.2	4.7	3.8	4.5	1.6
Myeloma	1.3	NA	0.9	1.0	0.2
Lymphoid leukaemia	0.9	NA	1.2	0.9	1.8
Myeloid leukaemia	1.8	NA	1.1	3.6	2.8
**All sites**	**114.5**	**116.2**	**101.4**	**104.3**	**166.8**

NA: Not available.
